# Approach or Avoidance: How Does Employees’ Generative AI Awareness Shape Their Job Crafting Behavior? A Sensemaking Perspective

**DOI:** 10.3390/bs15060789

**Published:** 2025-06-09

**Authors:** Yihang Yan, Xiaoqian Qu, Hongzhen Lei, Yao Geng

**Affiliations:** International Business School, Shaanxi Normal University, Xi’an 710119, China; yanyihang@snnu.edu.cn (Y.Y.); leihongzhen@snnu.edu.cn (H.L.); gengyao@snnu.edu.cn (Y.G.)

**Keywords:** GenAI awareness, job crafting, work passion, sensemaking, perceived CSR

## Abstract

Given the significant impact of Generative AI (GenAI) in the workplace, there are surprisingly few empirical studies examining how employees’ GenAI awareness shapes differently oriented job crafting behaviors. In organizations, understanding this is important because GenAI is unlikely to fully replace employees; instead, it requires them to adopt adaptive strategies to work alongside GenAI. If employees engage in avoidance crafting behavior, it could have negative consequences for the organization. Based on sensemaking theory, we develop a theoretical model to explore how employees’ GenAI awareness affects their job crafting behavior, as well as the mediating mechanisms and boundary conditions of its influence. Using self-evaluation data from 316 employees at three time points, the results of our hypothesis testing reveal that when employees perceive high internal Corporate Social Responsibility (CSR), their GenAI awareness triggers harmonious work passion and leads to approach job crafting; conversely, when employees perceive high external Corporate Social Responsibility, their GenAI awareness triggers obsessive work passion and leads to avoidance job crafting. Finally, the theoretical and empirical implications of our findings are discussed.

## 1. Introduction

**Figure 1:**
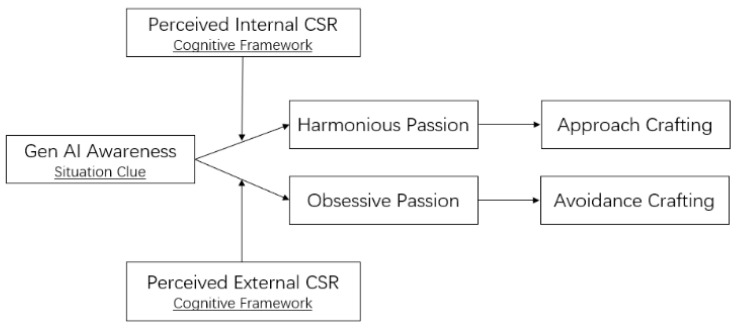
Theoretical model of the current research.

Generative AI (GenAI) represents the forefront of AI development and is profoundly reshaping the workforce by automating complex cognitive tasks, changing traditional workflows, and assisting with nearly all job functions (Eloundou et al., 2024). For example, GenAI accelerates the iterative design processes for creators (B. C. Lee & Chung, 2024), supports consultants in addressing realistic consulting tasks (Dell’Acqua et al., 2023), assists with software development tasks (Peng et al., 2023), and serves as a virtual customer service agent to support staff in resolving client issues (Brynjolfsson & Raymond, 2023). These technological changes require employees to continuously update their skills, redefine their job responsibilities to collaborate effectively with GenAI, and even re-evaluate their professional and organizational identities. In response, employees may proactively modify the boundaries of their job, which marks the beginning of job crafting behaviors (Wrzesniewski & Dutton, 2001). Job crafting is often categorized into two orientations: approach and avoidance (F. Zhang & Parker, 2019). Since these two forms lead to distinct work-related outcomes such as job engagement (J. Y. Lee & Lee, 2018) and job performance (Rudolph et al., 2017), they ultimately affect an organization’s ability to profit from the implementation of GenAI. Therefore, it is essential to understand how employees respond to the disruption and transformation brought by GenAI. Do they engage in approach crafting or avoidance crafting? Moreover, what underlying mechanisms and boundary conditions shape these divergent behavioral responses? The theoretical model is presented in Figure 1.

Scholars have previously conceptualized AI awareness (Brougham & Haar, 2018) and demonstrated that it can trigger both positive and negative workplace behaviors. With the substantial productivity gains driven by GenAI across industries (Noy & Zhang, 2023; Peng et al., 2023), approximately 80% of large organizations have implemented or plan to implement GenAI, accompanied by a notable increase in related investments over the past year (IBM, 2024). These developments have heightened employees’ awareness of GenAI. Building on prior research, we introduce the concept of GenAI awareness—defined as the employees’ subjective perception of GenAI’s impact on their careers, required skills, and potential threats. The uncertainty and complexity introduced by this profound technological revolution create an ideal context for sensemaking (Gioia & Chittipeddi, 1991). Accordingly, we adopt a sensemaking perspective to explore how GenAI awareness gives rise to differentiated job crafting behaviors. China’s high power distance and collectivist culture make employees particularly inclined to construct meaning and identity through organizational cues and actions (Weick, 1995). As a result, they are likely to interpret workplace changes—such as the implementation of GenAI—through the lens of perceived corporate social responsibility (CSR), that is, their beliefs about how the organization treats internal and external stakeholders (Brown et al., 2008; Weick et al., 2005; Weick, 1988). Perceived CSR consists of two distinct forms. Perceived internal CSR refers to employees’ perceptions of organizational efforts aimed at supporting employee well-being (O. Farooq et al., 2017) and may help to mitigate fears related to GenAI. In contrast, perceived external CSR reflects employees’ views of the organization’s actions toward external stakeholders (El Akremi et al., 2018). When overly emphasized, external CSR may be seen as a sign that the organization prioritizes image or profit over employee interests, thereby intensifying threats about GenAI. Based on sensemaking theory, we propose that the interaction between GenAI awareness and perceived internal/external CSR determines whether employees experience harmonious or obsessive passion, which in turn leads to distinct patterns of job crafting behavior.

We adopt the dualistic model of passion as the mediating mechanism. Harmonious passion arises from autonomous internalization of an identity, whereby individuals willingly engage in activities they enjoy. In contrast, obsessive passion results from controlled internalization, in which identity-related pressures compel individuals to engage in those same activities (Vallerand et al., 2003, p. 756). Prior studies on AI awareness have primarily focused on emotional and cognitive mechanisms (Teng et al., 2023) while largely overlooking work motivation—a critical and multifaceted factor in understanding employees’ behavioral responses. Passion has been both theoretically conceptualized and empirically validated as a fundamental motivational mechanism (Vallerand et al., 2003, 2007) and has proven effective in predicting employee behaviors such as job crafting (X. Zhang et al., 2023). Furthermore, the construction of perceived identity is a central component of the sensemaking process (Weick et al., 2005). The way individuals internalize work into their identities—either autonomously or in a controlled manner—is a key driver of work passion (Vallerand et al., 2003; Vallerand, 2015). Accordingly, work passion serves as a motivational bridge, linking GenAI awareness to job crafting through the partial process of identity construction. Building on this, we propose that the interaction between employees’ GenAI awareness and perceived CSR influences their approach or avoidance job crafting through harmonious or obsessive passion, respectively.

The main contributions are as follows: First, we contribute to the research on the impact of AI in the workplace by examining how employees’ GenAI awareness leads to different forms of job crafting behavior. In addition, we incorporate both approach and avoidance job crafting into a unified research framework to investigate the differentiated mechanisms through which GenAI awareness triggers these two orientations. By identifying the dual pathways of harmonious and obsessive passion, we offer new insights into how GenAI awareness can lead to either approach or avoidance crafting. In doing so, this study expands the empirical research on approach and avoidance job crafting and provides a more nuanced understanding of GenAI’s impact on employees’ workplace behaviors.

Second, this is the first study to apply a sensemaking perspective to explain the behavioral impact of GenAI. Drawing on sensemaking theory, we propose that perceived CSR serves as a critical interpretive framework through which employees make sense of GenAI. Unlike prior studies that conceptualize GenAI awareness as a job demand or a source of stress (He et al., 2024; Kang et al., 2023), we emphasize the interaction between GenAI awareness and perceived CSR in shaping whether employees perceive GenAI as a challenge or a hindrance. Furthermore, by distinguishing between internal and external CSR, we explain why employees develop different types of work passion and engage in distinct forms of job crafting. This study expands the understanding of the boundary conditions under which GenAI awareness influences employee behavior in organizations and contributes to research on how the interaction between individual and contextual factors affects job crafting.

Finally, this study adopts a motivational perspective by integrating the dualistic model of passion into the research framework, thereby enriching the understanding of the mediating mechanisms through which employees’ AI awareness influences workplace behavior. While prior research has shown that individuals interpret information and develop cognitions and attitudes during times of change, the motivational processes underlying sensemaking have been largely overlooked. By introducing work passion and using identity perception and internalization as a bridge, this study connects sensemaking theory with individual motivation and provides a better explanation for why employees interpret and respond differently to the same informational cues (e.g., GenAI). Moreover, it enhances our understanding of how and when work passion emerges, and how it subsequently influences workplace behaviors.

## 2. Theory Background and Hypothesis

### 2.1. Sensemaking Theory

Sensemaking often emerges from crises or unusual events, particularly when these changes involve people’s decisions, actions, or inactions (Sonenshein et al., 2014; Weick et al., 2005). On one hand, the application of GenAI transforms employees’ work content as well as their professional and organizational identities. This sudden technological disruption creates a powerful context for employees to engage in sensemaking (Sonenshein et al., 2014; Weick et al., 2005). On the other hand, given that individuals often attempt to rationalize their behaviors through the construction of meaning (Weick, 1995), this study adopts a sensemaking perspective to help organizations better understand the underlying reasons for the employees’ workplace behaviors.

According to Weick (1995), the sensemaking process involves three interconnected components: scanning, interpreting, and responding. Scanning refers to the collection of external information to identify events or issues relevant to the organization. Interpreting involves assigning meaning to this information based on one’s experiences, values, and knowledge. Responding entails taking action, shaped by the combined influence of scanning and interpretation.

As GenAI becomes increasingly integrated into the workplace, employees may perceive changes or even threats to their organizational identities, leading to GenAI awareness (scanning). This reflects an initial interpretation of the objective situational cue. According to sensemaking theory, the process begins not only with situational cues but also with the integration of cognitive frameworks (Brown et al., 2008; Weick, 1988). Given that GenAI-related changes directly affect employees’ personal interests, they are likely to draw on the organization’s attitudes and actions toward stakeholders (perceived CSR) to interpret how environmental shifts impact their current work and future career prospects (interpreting). As employees continuously integrate the objective impact of GenAI with their subjective perceptions of CSR, their understanding of how GenAI influences their identity deepens. This sensemaking process gives rise to either harmonious or obsessive passion, which in turn shapes distinct behavioral responses (responding). Specifically, when perceiving high-level internal CSR, employees are more likely to internalize new forms of work into their identities, fostering harmonious passion and engaging in approach job crafting. Conversely, when perceiving high-level external CSR, they may regard work as an external pressure, triggering obsessive passion and adopting avoidance job crafting behaviors.

### 2.2. GenAI Awareness, Perceived CSR, and Work Passion

#### 2.2.1. GenAI Awareness, Perceived Internal CSR, Harmonious Passion

In China’s internet and technology sector, CSR has gained increasing emphasis. Many leading firms now regularly publish CSR reports to enhance their public image and respond to growing institutional pressures (Hofman & Newman, 2014). Given their large workforce and heightened public scrutiny—particularly regarding labor practices such as the “996” work culture and overwork-related issues—these companies have also begun releasing internal CSR disclosures that focus on employee well-being and employer accountability. This contextual backdrop is essential for understanding how employees interpret CSR within their organizations. Internal CSR refers to the actions taken by organizations to meet employees’ expectations, actively promoting the fairness within the organization (such as improving employees’ health, well-being, and satisfaction), and ensuring job security as well as opportunities for growth and development (Brammer et al., 2007; Turker, 2009a). Harmonious passion stems from “the autonomous internalization of one’s identity, which leads individuals to engage in activities they enjoy” (Vallerand et al., 2003). It is a motivating force that enables individuals to invest their time and energy in these activities while maintaining control over their engagement.

When GenAI is introduced into the workplace, employees may develop GenAI awareness, accompanied by concerns about their relationship with work and their identity within the organization. In response, they tend to seek additional information to better understand the implications of GenAI. Organizations that prioritize internal stakeholders not only aim to benefit from GenAI adoption but also consider its impact on employees. Such organizations are more likely to provide adequate psychological and material support, including fair treatment, a supportive working environment, GenAI-related training, and career development opportunities. When the involvement of GenAI does not pose uncontrollable or harmful effects on employees, they tend to experience a strong sense of psychological safety and organizational identification (Tremblay et al., 2010). In such cases, employees perceive themselves as active agents in their work. They view GenAI’s partial task replacement not as a threat, but as an opportunity—freeing them from routine tasks and allowing more time to pursue self-growth through engaging in meaningful work, learning new skills, and tackling more complex problems. Over time, work becomes increasingly internalized as part of their identity, fostering a stronger sense of personal aspiration and professional purpose. In addition, internal CSR is typically based on voluntary actions taken by organizations and does not require material returns from employees (El Akremi et al., 2018; Flammer & Luo, 2017). As a result, employees experience less pressure to “give back to the organization” and are more motivated to engage proactively in their work out of a sense of reciprocity. Under high-level perceived internal CSR, the concerns associated with GenAI awareness are mitigated. Employees experience a greater sense of security and control, view task replacement as an opportunity rather than a threat, and develop a stronger identity recognition, promoting an unconditional intrinsic work motivation. These conditions collectively trigger harmonious passion (X. Zhang et al., 2023).

**Hypothesis** **1.** *When employees perceive high (vs. low) internal CSR, their GenAI awareness will be positively related to harmonious passion.*

#### 2.2.2. GenAI Awareness, Perceived External CSR, Obsessive Passion

External CSR refers to social responsibility actions towards local communities (charitable donations, community development investments, collaboration with non-governmental organizations, etc.), the natural environment (investments related to environmental protection), and consumers (commitments to consumer welfare, protection of consumer interests beyond the requirements of the law) (O. Farooq et al., 2014, 2017). Obsessive passion originates from “a controlled internalization of an activity in one’s identity that generates an internal pressure to engage in the activity” (Vallerand, 2015; Vallerand et al., 2019). This sense of pressure arises internally, from the nature of the work or interpersonal relationships, and is likely to cause individuals to lose control over the activity and feel compelled to participate.

Due to the socio-cultural and institutional context, Chinese employees tend to respond less positively to CSR practices targeted toward external stakeholders compared to employees in Western contexts and may even associate such efforts with job insecurity or resource diversion (D. Z. Ding et al., 2000; Farh et al., 2004). Within this context, organizations that prioritize external stakeholders are often perceived as primarily focused on enhancing their public image and reputation, ultimately seeking legitimacy and benefits from external audiences (Brammer et al., 2007; M. Farooq et al., 2014). Therefore, when employees realize that GenAI will replace certain aspects of their work, and at the same time perceive their organization as allocating limited resources to societal, environmental, or customer-focused initiatives rather than to employee support, they are likely to experience heightened insecurity. This perception reinforces the threatening aspect of GenAI awareness. Employees with high perceived external CSR may believe that their organization, in pursuit of rapid returns and technological breakthroughs through GenAI, is more inclined to recruit externally for GenAI-proficient talent rather than invest in the upskilling of existing employees. This interpretation results in identity threats and a growing sense of crisis among staff. Driven by the fear of being replaced, employees may engage in non-autonomous learning, pushing themselves to quickly acquire GenAI-related skills and adapt to new work demands. As GenAI-proficient employees become more efficient and productive (Jia et al., 2024), internal differentiation within the organization intensifies further, heightening the interpersonal competition. Thus, high perceived external CSR amplifies the threat component of GenAI awareness. The combined pressures of identity threat, non-autonomous learning, peer competition, and controlled, externally motivated adaptation may ultimately lead to obsessive passion (X. Zhang et al., 2023).

**Hypothesis** **2.** *When the perception of external CSR is high, employees’ GenAI awareness will trigger obsessive passion.*

### 2.3. Approach Crafting or Avoidance Crafting? Employees’ Behavioral Responses to Harmonious and Obsessive Work Passion

When employees construct identity perceptions by integrating their awareness of GenAI’s application in the workplace with their perceptions of CSR, they begin to ask themselves, “What should I do next?” (Weick et al., 2005). Building on the definitions by Tims et al. (2012, 2013), approach job crafting involves increasing structural job resources (e.g., initiating new ways of working), increasing social job resources (e.g., asking for feedback from colleagues and/or supervisors), and increasing challenging job demands (e.g., engaging in interesting projects). In contrast, avoidance job crafting focuses on decreasing hindering job demands (e.g., avoiding difficult tasks and social interactions) (Bipp & Demerouti, 2015; Bruning & Campion, 2018). We propose that harmonious passion triggers approach job crafting, while obsessive passion triggers avoidance job crafting.

Evidence suggests that individuals with high levels of harmonious passion display greater curiosity and flexibility in exploring new ways and creative methods to reshape their work tasks and social relationships (Liu et al., 2011; Shalley et al., 2004). Therefore, we propose that harmonious passion triggers approach job crafting. Specifically, employees with harmonious passion tend to have a strong sense of identity, which motivates them to build and strengthen workplace relationships. They may proactively establish connections with colleagues and supervisors, seek advice and feedback, and participate in both work-related and informal social interactions to expand their interpersonal networks. In addition, their high intrinsic interest in work leads them to express and expand their interests through approach crafting behaviors, such as actively learning new knowledge or seeking novel and interesting tasks (Bindl et al., 2019; X. Zhang et al., 2023). This enables them to continuously experience personal growth at work (Parker et al., 2010). Finally, employees with harmonious passion tend to integrate their work into their self-identity. This internalized identity motivates them to continuously adjust and personalize their tasks to better align with their values and self-identity (Gagné & Deci, 2005; Sheldon & Elliot, 1999; Vallerand, 2015), thereby enhancing the meaning of their work and the person–job fit.

**Hypothesis** **3.** *Harmonious passion is positively related to approach job crafting.*

Avoidance job crafting refers to employees proactively narrowing their cognitive and task boundaries by reducing work tasks or minimizing social interactions with colleagues and supervisors (Bipp & Demerouti, 2015; Lopper et al., 2024). Although some studies have found that obsessive passion is associated with both positive outcomes (e.g., increased time investment, positive affect) and negative outcomes (e.g., rumination, emotional exhaustion) (Curran et al., 2015; Pollack et al., 2020), this study focuses on its potential downsides in the context of GenAI-driven workplace change. In particular, within high-pressure, overtime-intensive environments like internet and technology firms, the negative effects of obsessive passion may be amplified. Employees with obsessive passion often experience a sense of control and compulsion, along with negative emotional states and reduced well-being (Vallerand, 2015). Such conditions can result in significant resource depletion (Pollack et al., 2020), leaving employees without the energy or motivation to engage deeply with GenAI. Instead, they may resort to learning the basic functions of GenAI to meet immediate job demands. Moreover, employees driven by obsessive passion typically lack intrinsic motivation. Their engagement is primarily driven by external pressures—such as a fear of replacement or the desire to gain managerial approval—rather than a genuine interest in learning or self-improvement. As a result, they may rely on GenAI to complete routine tasks quickly in order to appear productive but lack the motivation to explore more advanced applications or take on complex challenges. They are also less likely to seek additional responsibilities or proactively request feedback from supervisors. Finally, the interpersonal pressure associated with obsessive passion may reduce communication and collaboration with colleagues. These withdrawal behaviors, such as avoiding task expansion or feedback-seeking, and reducing job responsibilities and engagement levels, are indicative of avoidance crafting (C. H. Wu & Li, 2016).

**Hypothesis** **4.** *Obsessive passion is positively related to avoidance job crafting.*

### 2.4. A Mediated Moderation Model

The core purpose of employees using a cognitive framework (perceived internal/external CSR) to interpret objective situational cues (GenAI) in sensemaking is to find an appropriate identity in a dynamically changing environment. However, different cognitive frameworks lead to varying interpretations of the same objective cue (Balogun et al., 2015), driving the harmonious or obsessive work passion and ultimately leading to different orientations of job crafting behaviors. In this section, we propose two mediated moderation models: the interaction between employees’ GenAI awareness and perceived internal CSR affects approach job crafting through harmonious passion, and the interaction between employees’ GenAI awareness and perceived external CSR affects avoidance job crafting through obsessive passion.

When employees perceive a high level of internal CSR, they experience a strong sense of psychological safety and organizational support. They view GenAI’s partial replacement of their tasks as an opportunity for self-improvement, which fosters unconditional intrinsic motivation and leads to the internalization of work into their personal identity, ultimately giving rise to harmonious passion. Under the influence of harmonious passion, individuals are more focused on enjoyment and personal growth (Vallerand, 2015). Driven by intrinsic motivation and a sense of autonomy, they seek to align their work with their personal interests and values—making job crafting an effective strategy for achieving such alignment. This pursuit of meaning and self-development motivates them to actively acquire GenAI-related knowledge, seek feedback from supervisors and colleagues, and proactively adjust their work in flexible and intentional ways, thereby enhancing their efficiency and problem-solving capabilities.

When employees perceive a high level of external CSR, they may interpret it as the organization prioritizing external stakeholders over employee welfare, including their physical and mental well-being. In such cases, the career uncertainty and complexity caused by GenAI would be magnified (Vallerand et al., 2003; H. J. Wang et al., 2015). Employees may internalize GenAI-related work as a form of external pressure, arising from a heavy workload, peer competition, and the need to secure their position within the organization. Under such conditions, they are more likely to develop obsessive passion. When experiencing high levels of obsessive passion, employees tend to develop controlled and externally-driven motivation, which can lead to negative perceptions of their work environment. In these contexts, avoidance crafting may serve as a coping mechanism to release internal tension and maintain emotional well-being. Moreover, due to a perceived loss of autonomy and control over their work, these employees may withdraw from proactive activities and instead engage in passive or defensive behaviors (G. Wu et al., 2023). Faced with increasing pressure from work and peer competition, they may intentionally reduce their task and social boundaries as a means to conserve personal resources and protect themselves from additional challenges in the workplace.

**Hypothesis** **5.** *The interaction between employees’ GenAI awareness and their perceived internal CSR significantly and positively influences approach job crafting through harmonious passion.*

**Hypothesis** **6.** *The interaction between employees’ GenAI awareness and their perceived external CSR significantly and positively influences avoidance job crafting through obsessive passion.*

## 3. Methods

### 3.1. Participants and Procedures

We recruited 315 participants through Credamo, an online survey platform known for its capacity to collect large-scale, high-quality data in China (Buhrmester et al., 2011). To ensure relevance to the research context, we restricted the sample to employees in large internet and technology companies—industries among the earliest adopters of GenAI and where it has significantly transformed work processes.

The study adopted a three-wave design, with a three-week interval between each wave, to strengthen causal inferences and mitigate common method bias. At Time 1, participants were asked two screening questions: (1) “Is your company a large internet or technology firm (e.g., with over 1000 employees or recognized as an industry leader)?” and (2) “Do you use GenAI tools at work (e.g., ChatGPT, Kimi, Wenxin Yiyan, Doubao, etc., including any version you may have access to)?”. Respondents who answered “No” to either question were automatically directed to the end page with a thank-you message, since the survey was specifically targeting this population. Those who answered “Yes” to both questions proceeded to complete the Time 1 survey, in which participants reported their demographic information (gender, age, and education level), frequency of GenAI use, AI awareness, and perceived CSR. At Time 2, participants who completed the first wave were invited to assess their level of work passion (harmonious and obsessive). At Time 3, the same respondents evaluated their job crafting behaviors (approach and avoidance). Written informed consent was obtained from all participants.

As the Credamo platform requires participants to complete all items before submission, there were no missing data. The platform also enables researchers to monitor completion times, preview responses, and reject unsatisfactory submissions. Prior to accepting responses, we screened all entries and excluded those with implausibly short completion times, highly patterned answer selections, or responses indicating “never” for GenAI usage frequency. A total of 600 surveys were distributed at Time 1, and 316 valid responses were retained through Time 3. Although this design does not constitute a full time series analysis, the temporal separation of key constructs enhances the validity of the proposed mediation pathways and reduces concerns about simultaneity or reverse causation. Descriptive statistics for the final sample are presented in Table 1.

To evaluate the adequacy of our sample size (N = 316), we conducted post hoc power analyses using G*Power 3.1 (Faul et al., 2009). Power was calculated for four key hypothesized paths in the model. Table 2 summarizes the standardized regression coefficients (β), estimated R^2^, effect sizes (f^2^), and corresponding statistical power values for each focal path. The estimated power values were >0.99, 0.98, 0.85, and 0.61, respectively. Three of the four paths exceeded the conventional threshold of 0.80 (Cohen, 1988), indicating adequate power to detect the primary effects. The relatively lower power for the interaction between GenAI awareness and external CSR (≈0.61) reflects the typically small effect size of moderation effects observed in field studies (Aguinis et al., 2005). Overall, the results suggest that the current sample size is sufficient to support the main effects and key moderated mediation pathways examined in this study.

### 3.2. Measures

The measures used in this study were adopted from the existing literature. All English-based measures were translated into Chinese using the “translation/back-translation” procedures (Brislin, 1986) by a panel of bilingual experts. A Likert-type scale ranging from 1 (strongly disagree) to 5 (strongly agree) was used for all questions.

GenAI Awareness. We adopted four items from Brougham and Haar (2018) to measure employees’ GenAI awareness, which have been used in previous studies (Kong et al., 2021; Li et al., 2019).We made slight wording modifications on the basis of the original items to better fit the context of our research. An example item is “Given that GenAI is being widely used in the workplace, I am concerned about my future in this industry” (α = 0.732).

Employees’ Perceived CSR. In line with prior studies on CSR in the Chinese context (e.g., Hofman & Newman, 2014; Tian et al., 2015), we adopted Turker’s (2009b) scale, which captures employees’ perceptions of their organization’s responsibility toward various stakeholder groups. Perceived internal CSR was measured using a six-item subscale from the “CSR to employees” subscale. An example item for perceived internal CSR is “The management of my organization is primarily concerned with employees’ needs and wants” (α = 0.785). Perceived external CSR was measured using a 6-item subscale from the “CSR to social and non-social stakeholders” (i.e., community and environment) subscale. An example item is “My company makes adequate contributions to charities” (α = 0.732).

Passion for Work. We measured task interdependence using the 8-item passion scale developed by Vallerand et al. (2003). We selected the top four items with the highest factor loadings from the original scale. Harmonious passion was measured using four items; an example item is “My work reflects the qualities I like about myself” (α = 0.739). Obsessive passion also was assessed with four items, including “The urge is so strong, I can’t help myself from doing my work” (α = 0.857).

Job Crafting. To measure approach crafting and avoidance crafting, we adopted the job crafting scale developed by Tims et al. (2012), which has been used in several studies (e.g., Harju et al., 2021; A. J. Xu et al., 2024). Approach crafting (α = 0.809) involves three dimensions, including increasing structural job resources (5 items, e.g., “I try to develop my capabilities”), increasing social job resources (5 items, e.g., “I ask my supervisor to coach me”), and increasing challenging job demands (5 items, e.g., “When an interesting project comes along, I offer myself proactively as project co-worker”). Avoidance crafting (α = 0.706) was measured by decreasing hindering job demands (6 items, e.g., “I make sure that my work is mentally less intense”).

Control Variables. Prior literature has proven that demographic variables including gender, age, and educational level can influence both employees’ passion for work and job crafting. In addition, the frequency of using GenAI would affect their familiarity with GenAI and also has an impact on GenAI awareness.

### 3.3. Test of Measurement Models

Confirmatory factor analysis (CFA) was conducted using Mplus 8.3 to examine the discriminant validity of all latent variables. To balance the relationship between the sample size and the number of estimated parameters, we adopted an item parceling approach. The multi-dimensional construct approach crafting was parceled using the internal consistency method by grouping items within the same subdimension, while the remaining constructs (except GenAI awareness) were parceled using the balanced approach (Y. Wu & Wen, 2011; Little et al., 2002). Perceived internal CSR, perceived external CSR, and avoidance crafting were each represented by three parallel indicators.

As shown in Table 3, the results of the CFA reveal that the eight-factor model had better fit indices compared to the alternative models (X^2^/df = 1.849; CFI = 0.951; TLI = 0.938; RMSEA = 0.052; SRMR = 0.044), which indicated that our model had good discriminant validity.

To control the potential issue of common method bias (CMB), we used a procedural method and a statistical control method. Procedurally, data were collected at three separate time points to reduce method bias. Statistically, we employed two approaches. First, Harman’s single-factor test was conducted via confirmatory factor analysis (CFA), constraining all items to load on a single latent factor. The model explained 29.81% of the variance, much below the 50% threshold, suggesting that CMB was not a major concern. Second, we applied the unmeasured latent method factor control (ULMC) by adding an error variable factor to the seven-factor model. Model comparisons showed minimal changes in fit indices (ΔCFI = 0.031, ΔTLI = 0.036, ΔRMSEA = 0.020), all below the recommended threshold of 0.05 (Bagozzi & Yi, 1990; Podsakoff et al., 2012), indicating that CMB did not have a serious impact on this study.

## 4. Results

The means, standard deviations, and correlation coefficients of the variables, as presented in Table 4, fall within acceptable ranges. To assess potential multicollinearity, we calculated the variance inflation factors (VIFs) for all predictor variables (Hair et al., 2010). The VIF values were well below the commonly accepted threshold of 5—specifically, 1.49 for GenAI awareness, 1.64 for perceived internal CSR, and 1.23 for perceived external CSR—indicating that multicollinearity was not a concern in the regression models (Hair et al., 2010). Among the control variables, GenAI usage frequency was significantly associated with several focal constructs, including GenAI awareness (β = −0.270, *p* < 0.01), perceived internal CSR (β = −0.311, *p* < 0.05), perceived external CSR (β = −0.174), harmonious passion (β = −0.270 *, *p* < 0.05), and approach crafting (β = −0.353, *p* < 0.01). These associations were statistically controlled in all regression models.

To provide an overview of the hypothesized relationships and their empirical support, Table 5 presents a summary of all hypotheses tested in this study.

**Figure 2:**
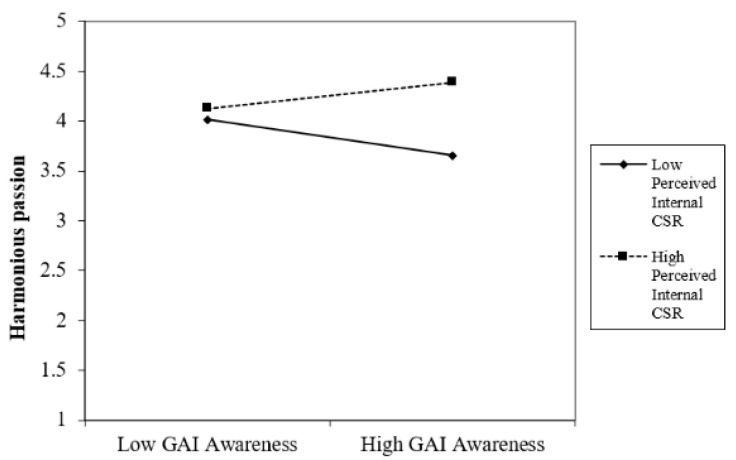
Interactive effect of GenAI × INCSR.

Hypothesis 1 proposed that the employees’ GenAI awareness would be positively related to harmonious passion only under a high level of perceived internal CSR. As shown in Table 6, there was no significant main effect of the employees’ GenAI awareness on their harmonious passion (β = −0.018, *p* = 0.786). However, our results showed that the employees’ GenAI awareness interacted with perceived internal CSR to predict harmonious passion (R^2^ = 0.200, β = 0.532, *p* < 0.001). We plotted this significant interactive effect in Figure 2. Simple slope t tests indicated that at a lower level of perceived internal CSR (−1 SD), there was a negative relationship between the employees’ GenAI awareness and harmonious work passion (b = −0.350, t = −17.738, *p* < 0.001). A positive correlation was observed at the higher level (+1 SD) of employees perceived internal CSR, when employees’ GenAI awareness was positively related to harmonious passion (b = 0.256, t = 7.351, *p* < 0.001), thus Hypothesis 1 was supported.

**Figure 3:**
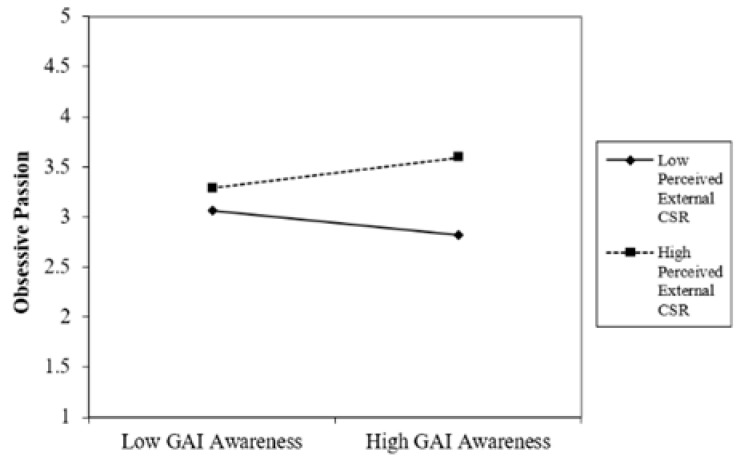
Interactive effect of GenAI × EXCSR.

Hypothesis 2 proposed that there is a positive correlation between the employees’ GenAI awareness and obsessive work passion only when the perceived external CSR is high. As reported in Table 6, the main effect of employees’ GenAI awareness on obsessive work passion is not significant (β = 0.116, *p* = 0.317). However, the interaction between the employees’ GenAI awareness and perceived external CSR predicted obsessive work passion (R^2^ = 0.086, β = 0.519, *p* < 0.01). We plotted this interaction result in Figure 3. The resulting simple slope t test showed that, under the condition of higher-level (+1 SD) perceived external CSR, employees’ GenAI awareness was positively related with obsessive passion (b = 0.295, t = 1.8354, *p* < 0.05). This positive relationship turned negative under the condition of lower-level (−1 SD) perceived external CSR (b = −0.240, t = −3.208, *p* = 0.001). Hypothesis 2 was thus supported.

Hypothesis 3 proposed that the employees’ harmonious work passion would be positively related to their approach job crafting. As shown in Table 6, employees’ harmonious passion was positively related to approach crafting (R^2^ = 0.216, β = 0.277, *p* < 0.001). Surprisingly, we found that harmonious passion also positively predicted avoidance crafting (β = 0.177, *p* < 0.001); we will explain this finding later. Hypothesis 3 was thus supported.

Hypothesis 4 proposed that the employees’ obsessive work passion would be positively related to their avoidance job crafting. As shown in Table 6, employees’ obsessive passion was positively related to avoidance crafting (R^2^ = 0.123, β = 0.166, *p* < 0.001), but was not significantly related to approach crafting (β = 0.024, *p* = 0.344). Hypothesis 4 was thus supported.

Hypothesis 5 proposed that the indirect effect of the interaction between employees’ GenAI awareness and perceived internal CSR on approach job crafting was mediated by harmonious work passion. Under the condition of higher-level perceived internal CSR, the positive indirect effect on approach job crafting would be stronger. As reported in Table 7, the results showed that the interaction of employees’ GenAI awareness and perceived internal CSR had a significantly positive indirect effect on their approach crafting (ρ = 0.112, *p* < 0.01, 99% CI [0.032, 0.236]) via harmonious passion. Thus, Hypothesis 5 was supported. Due to the positive relationship between harmonious work passion and approach job crafting we found previously, we further tested the interaction of employees’ GenAI awareness and perceived internal CSR on avoidance job crafting, which was also mediated by harmonious work passion (ρ = 0.075, *p* < 0.01, 95% CI [0.011, 0.163]).

Hypothesis 6 proposed that the indirect effect of the interaction between employees’ GenAI awareness and perceived external CSR on avoidance job crafting was mediated by obsessive work passion. Under the condition of higher-level perceived external CSR, the positive indirect effect on avoidance job crafting would be stronger. As reported in Table 7, the results showed that the interaction of employees’ GenAI awareness and perceived external CSR had a significantly positive indirect effect on their avoidance crafting (ρ = 0.085, *p* < 0.01, 99% CI [0.012, 0.184]) via obsessive passion. Thus, Hypothesis 6 was supported.

## 5. Discussion

Drawing on sensemaking theory, we propose that the organizational application of GenAI represents a major situational cue that prompts employees to engage in sensemaking. In this process, employees interpret the potential impact of GenAI on their roles and identities by integrating it with their perceived CSR, thereby rationalizing their subsequent behavioral responses. Following the scan—interpret—respond sequence of sensemaking (Weick, 1995), we develop a dual-pathway model to explain how employees’ awareness of GenAI leads to distinct forms of work passion and job crafting behavior.

Our findings support these hypotheses. The results suggest that perceived internal CSR serves as a positive interpretive frame through which employees make sense of GenAI. When employees perceive a high level of internal CSR, they are more likely to view GenAI as an opportunity for self-development or career transformation. This interpretation enables them to autonomously internalize GenAI-related work, which in turn fosters harmonious passion and leads to approach job crafting. Conversely, under high perceived external CSR, employees may interpret GenAI as a threat to their professional identity. The combination of heightened external pressure and controlled motivation triggers obsessive passion, ultimately resulting in avoidance-oriented job crafting.

Interestingly, we also observed a secondary pathway in which harmonious passion was associated with avoidance crafting. This suggests that high levels of harmonious passion may lead employees to strategically disengage from certain demands in order to conserve personal resources or maintain a work-life balance.

### 5.1. Theoretical Implications

Our study makes several theoretical contributions to the relevant literature.

First, we introduce the concept of GenAI awareness and examine how employees’ GenAI awareness leads to different forms of job crafting behavior. Compared to traditional AI, GenAI has had a far greater impact on the workforce by creating new job opportunities, transforming work processes, and even introducing entirely new roles. Researchers have found that human-GenAI collaboration can improve overall work efficiency (Noy & Zhang, 2023; Peng et al., 2023). However, it also poses challenges, such as performance decline caused by an over-reliance on GenAI and long-term skill degradation. Moreover, when tasks exceed GenAI’s capabilities, reliance on it may lead to reduced output quality (Dell’Acqua et al., 2023). Thus, the application of GenAI in the workplace places greater emphasis on human-GenAI collaboration, offering employees more autonomy in deciding how to engage with their work. As a result, job crafting behaviors triggered by GenAI may be either approach-oriented or avoidance-oriented. While recent studies (e.g., He et al., 2024; Kang et al., 2023) have investigated the impact of AI awareness on job crafting, they conceptualized job crafting as a single construct and did not consider the dual nature of approach versus avoidance strategies. By constructing a dual-pathway model of how GenAI awareness shapes employees’ job crafting behaviors, our study provides a more comprehensive perspective on the impact of GenAI awareness and extends the literature on the antecedents of both approach and avoidance job crafting.

Second, our study adopts a sensemaking theoretical perspective to explore how employees perceive GenAI awareness—either as a negative impediment or a positive challenge—and how they proactively respond to its implementation. Most previous studies have preemptively framed AI as a job demand or obstacle through resource perspectives and pressure perspectives. Using Conservation of Resources Theory (H. T. Wang & Xu, 2023; G. Xu et al., 2023), the JD-R model (He et al., 2024; Liang et al., 2022), Self-Determination Theory (Tan et al., 2023), and Cognitive Appraisal Theory (L. Ding, 2021; Zhao et al., 2023), these studies describe how employees’ AI awareness, perceived as a threat to their career development or organizational status, triggers demands, stress, emotional exhaustion, and insecurity, ultimately leading to negative outcomes. However, whether in real organizational contexts or empirical studies, findings highlight diverse employee attitudes and behavioral responses toward GenAI; some perceive it as a hindrance, while others embrace it as a challenge. Given the workplace disruption brought by GenAI, employees are likely to engage in sensemaking. Drawing on sensemaking theory, this study positions perceived CSR as a cognitive framework through which employees interpret GenAI-driven changes. We demonstrate how variations in perceived CSR shape employees’ interpretations of GenAI, triggering distinct forms of work passion and job crafting behaviors. To our knowledge, this is the first study to examine the impact of GenAI from a sensemaking perspective, thereby extending the application of sensemaking theory to technological change. It also validates the role of perceived CSR as an effective cognitive frame in the sensemaking process. This perspective complements existing research by integrating individual and contextual factors to explain employees’ proactive responses—specifically, approach and avoidance job crafting.

Third, our study expands the understanding of how and when work passion arises and influences workplace behaviors; through work passion, we connect sensemaking theory with motivation. Most previous studies have mainly focused on how employees’ AI awareness determines work-related behaviors through a single mediating mechanism, emphasizing emotional, cognitive, and behavioral perspectives with limited attention to the role of work motivation. However, motivation represent a more fundamental psychological mechanism compared to emotions and cognition (Liu et al., 2011), serving as a prerequisite for stimulating employees’ proactive behaviors. We contribute to filling this gap by examining the relationship between work passion and different behavioral strategies. According to sensemaking theory, the identity construction triggered by GenAI awareness and perceived CSR will continue to unfold. As a result, employees will experience different types of work passion during the process of internalizing work into their personal identity, which will lead to approach or avoidance job crafting. We found that obsessive passion is positively correlated with avoidance job crafting, while harmonious passion positively predicts approach job crafting and is also associated with avoidance job crafting. This is because employees with high levels of harmonious passion are better at balancing their work and personal lives. When faced with significant life events or work-related stress, they might reduce their engagement with work to conserve personal resources. Overall, this study deepens our understanding of GenAI-induced work motivations and their differential effects on workplace behaviors and also enriches the research on the antecedents of job crafting.

### 5.2. Practical Implications

This study also offers valuable practical implications for organizations. Our findings suggest that the integration of GenAI into the workplace is more likely to trigger and amplify employees’ job crafting behaviors—both approach and avoidance—compared to tasks without AI involvement. Therefore, organizations promoting or preparing to deploy GenAI should pay close attention to employees’ GenAI awareness. Employees with a positive attitude toward GenAI tend to actively learn how to use it, collaborate with it to generate new ideas, and engage in open communication with supervisors and peers to explore innovative applications. Conversely, those with a more negative attitude may rely on GenAI primarily to reduce their workload in existing tasks, thus engaging in avoidance-oriented crafting. Therefore, organizations should not only focus on using GenAI to cut costs and improve efficiency but also pay close attention to how employees perceive and respond to this technological change. For instance, companies can host open discussions to gauge employee sentiments about GenAI, offer targeted training programs, and create opportunities for both vertical and peer-level communication to encourage approach-oriented job crafting.

In addition, we found that employee’ perceived CSR plays a crucial role in shaping the impact of their GenAI awareness on their work passion and crafting behaviors. When GenAI is integrated into work, employees who perceive internal CSR are more likely to experience harmonious passion, leading to approach job crafting, while those perceiving external CSR are more likely to experience obsessive passion, resulting in avoidance job crafting. To manage this dynamic effectively, organizations should ensure that employees feel genuinely supported and valued during technological transformations. Organizations can offer GenAI-related training programs, invite experts to provide personalized career counseling and transition planning, and grant employees greater autonomy in work design to foster a sense of control and respect. Moreover, when organizations engage in external CSR initiatives, it is critical to avoid sending signals that employee well-being is being neglected. To mitigate this risk, organizations should clearly communicate the purpose and relevance of external CSR activities—explaining how these initiatives ultimately benefit both society and employees. For instance, external CSR can enhance the organization’s long-term stability and reputation, which in turn contributes to job security and a more supportive work environment.

Moreover, our research suggests that while harmonious passion can lead to approach job crafting, it may also result in avoidance job crafting. Obsessive passion, on the other hand, exclusively leads to avoidance job crafting. Hence, organizations should aim to minimize obsessive passion while carefully managing overly high levels of harmonious passion. To achieve this, managers can foster a psychologically safe environment and offer employees a sense of autonomy and competence—two key factors that promote autonomous motivation and mitigate burnout risks (Parker et al., 2006; Wrzesniewski & Dutton, 2001). For example, job redesign practices that align tasks with employees’ values and interests, along with the meaningful recognition of employee efforts, can reinforce sustainable harmonious passion. In addition, organizations can provide training or coaching to help employees reflect on their passion levels, set personal boundaries, and adopt adaptive work strategies. These interventions can help employees maintain high engagement without overextending themselves.

### 5.3. Limitations and Future Directions

Our research provides valuable insights into the related literature but also has some limitations, which we hope will inspire further research in this area.

Firstly, while this study reveals the dual pathways through which employees’ GenAI awareness influences job crafting behaviors under different levels of perceived CSR, it does not account for the potential moderating effects of other variables. Specifically, we did not consider how individual characteristics (e.g., personality traits, career orientation), team-level factors (e.g., transformational leadership), or contextual variables (e.g., team members’ GenAI capabilities) might shape these relationships. Future research could incorporate both individual traits and contextual influences to more comprehensively identify the boundary conditions under which GenAI awareness affects job crafting behavior.

Secondly, in terms of data collection, although we employed a multi-source, multi-wave design to minimize concerns about common method bias and measurement bias, our study could not capture the temporal evolution of work passion and job crafting. Future research should explore how these dynamics unfold over time when employees collaborate with GenAI. For instance, scholars could investigate when harmonious passion becomes excessive and gradually shifts toward obsessive passion, or how harmonious passion reinforces a virtuous cycle of approach job crafting. Additionally, as all data were collected in China, the country’s unique cultural characteristics may influence how employees interpret CSR and respond to technological change. Future research should examine the interaction between GenAI awareness and perceived CSR in different national and cultural contexts to enhance the generalizability of the findings.

Lastly, while this study adapted an existing AI awareness scale to the context of GenAI, we acknowledge that the adapted measure has not undergone full psychometric validation specific to GenAI applications. Although our confirmatory factor analysis indicated acceptable reliability and model fit, future research should further assess the construct validity of the GenAI awareness scale. This could be achieved through additional validation techniques, such as convergent/discriminant validity testing or measurement invariance analysis across industries or cultural settings. Establishing a validated and domain-specific scale would strengthen the robustness and generalizability of the findings in this emerging research area.

## 6. Conclusions

This study introduces the construct of GenAI awareness and highlights that, compared to traditional workplace contexts, the new knowledge, skills, flexible tasks, and career development opportunities brought by GenAI are more likely to stimulate employees to engage in broader job crafting behaviors. Unlike prior studies that primarily focus on cognitive or emotional mechanisms, we adopt a motivational perspective by incorporating the dualistic model of passion to explain and validate the dual-pathway effects of GenAI awareness on employee job crafting. Most importantly, this is the first study to apply sensemaking theory to investigate the workplace impact of GenAI. We demonstrate that employees interpret GenAI awareness in conjunction with perceived internal/external CSR, which in turn shapes their experience of harmonious or obsessive passion—ultimately leading to approach or avoidance job crafting. These insights offer a novel theoretical lens for understanding how employees respond to AI-driven workplace transformations.

## Figures and Tables

**Table 1:** Demographic information (N = 316).

Items	Options	Sample	Percentage
Gender	Male	111	35.13%
	Female	205	64.87%
Age	20–30	164	51.90%
	31–40	127	40.19%
	41–50	9	2.80%
	51–60	16	5.10%
Education level	Junior College and Below	19	6.01%
	Bachelor’s Degree	214	67.72%
	Master’s Degree	79	25%
	Doctorate	4	1.27%
Years of Service	1–5 years	207	65.51%
	5–10 years	65	20.57%
	10–20 years	30	9.49%
	More than 20 years	14	4.43%
Frequency of Using GenAI	Every day	136	43.04%
	Every week	136	43.04%
	Every month	13	4.11%
	Never	0	0%

**Table 2:** Post hoc power analysis for four key paths.

Hypothesis	Beta (β)	Estimated R^2^	f^2^	Power (N = 316)
H1: GenAI × Internal CSR → Harmonious Passion	0.532	0.283	0.395	>0.99
H2: GenAI × External CSR → Obsessive Passion	0.101	0.01	0.01	≈0.61
H3: Harmonious Passion → Approach Crafting	0.277	0.077	0.083	≈0.98
H4: Obsessive Passion → Avoidance Crafting	0.166	0.028	0.028	≈0.85

**Table 3:** Results of confirmatory factor analysis.

Model	Factor	X^2^/df	CFI	TLI	RMSEA	SRMR
1. Seven-factor Model	GAIA; INCSR; EXCSR; HP; OP; AP; AV	1.849	0.951	0.938	0.052	0.044
2. Six-factor Model	GAIA; INCSR + EXCSR; HP; OP; AP; AV	2.733	0.896	0.873	0.074	0.059
3. Six-factor Model	GAIA; INCSR; EXCSR; HP + OP; AP; AV	3.556	0.847	0.812	0.090	0.077
4. Six-factor Model	GAIA; INCSR; EXCSR; HP; OP; AP + AV	2.692	0.899	0.876	0.073	0.066
5. Five-factor Model	GAIA; INCSR + EXCSR; HP+OP; AP; AV	4.301	0.795	0.757	0.102	0.084
6. Five-factor Model	GAIA; INCSR + EXCSR; HP; OP; AP + AV	3.503	0.845	0.816	0.089	0.076
7. Five-factor Model	GAIA; INCSR; EXCSR; HP + OP; AP + AV	4.235	0.800	0.762	0.101	0.086
8. Four-factor Model	GAIA; INCSR + EXCSR; HP + OP; AP + AV	4.965	0.748	0.708	0.112	0.093

Note: N = 316. GAIA represents GenAI awareness, INCSR represents perceived internal CSR, EXCSR represents perceived external CSR, HP represents harmonious passion, OP represents obsessive passion, AP represents approach crafting, AV represents avoidance crafting.

**Table 4:** Descriptive statistics and correlations between the study variables.

Variables	1	2	3	4	5	6	7	8	9	10	11	Mean	SD
1. Gender (T1)												1.649	0.4781
2. Age (T1)	−0.105											1.611	0.7748
3. Education Level (T1)	0.117 *	−0.194 **										2.215	0.5617
4. Tenure (T1)	−0.105	0.750 **	−0.195 **									1.528	0.8409
5. Frequency of Using GenAI (T1)	−0.025	0.248 **	−0.143 *	0.195 **								1.756	0.8091
6. GenAI Awareness (T1)	−0.008	0.182 **	−0.065	0.108	−0.270 **							4.168	0.5137
7. Perceived Internal CSR (T1)	−0.040	0.086	−0.046	0.029	−0.311 **	0.566 **						4.115	0.5693
8. Perceived External CSR (T1)	−0.034	0.040	−0.090	0.059	−0.174 **	0.312 **	0.425 **					4.089	0.5158
9. Harmonious Passion (T2)	−0.030	0.020	−0.011	0.005	−0.270 **	0.085	0.302 **	0.237 **				4.134	0.5691
10. Obsessive Passion (T2)	−0.018	−0.045	−0.018	−0.006	0.070	0.016	0.006	0.210 **	−0.013			3.236	0.9738
11. Approach Crafting (T3)	0.051	0.068	0.052	0.031	−0.353 **	0.678 **	0.500 **	0.372 **	0.345 **	0.046		4.184	0.4559
12. Avoidence Crafting (T3)	0.024	0.045	−0.005	0.038	−0.093	0.268 **	0.198 *	0.199 **	0.196 **	0.316 **	0.309 **	4.045	0.5122

Note: N = 316; * *p* < 0.05; ** *p* < 0.01.

**Table 5:** Hypotheses summary.

Hypothesis	Path/Statement
H1	GenAI × Internal CSR → Harmonious Passion
H2	GenAI × External CSR → Obsessive Passion
H3	Harmonious Passion → Approach Crafting
H4	Obsessive Passion → Avoidance Crafting
H5	GenAI × Internal CSR → Harmonious Passion → Approach Crafting
H6	GenAI × External CSR → Obsessive Passion → Avoidance Crafting

**Table 6:** Results of hypotheses testing.

	Dependent Variables							
	Harmonious Passion		Obsessive Passion		Approach Crafting		Avoidance Crafting	
Control Variables	B	SE	B	SE	B	SE	B	SE
Gender	−0.030	0.066	−0.042	0.116	0.034	0.033	0.036	0.074
Age	0.078	0.063	−0.168	0.111	−0.021	0.032	0.157 *	0.070
Education Level	0.037	0.057	−0.017	0.101	0.047	0.029	−0.036	0.064
Tenure	−0.016	0.056	0.073	0.099	0.013	0.029	−0.044	0.063
Frequency of Using GenAI	−0.212 ***	0.042	0.127	0.075	−0.038	0.022	−0.044	0.048
**Predicting Variables**								
GenAI Awareness	−0.018	0.065	0.116	0.116				
Harmonious Passion					0.277 ***	0.042	0.177 ***	0.050
Obsessive Passion					0.024	0.025	0.166 ***	0.028
**Interactions**								
GenAI Awareness × Perceived Internal CSR	0.532 ***	0.120	0.087	0.228				
GenAI Awareness × Perceived External CSR	0.101	0.109	0.519 **	0.190				
**R^2^**	0.200		0.086		0.216		0.123	

Note: N = 316; * *p* < 0.05; ** *p* < 0.01; *** *p* < 0.001.

**Table 7:** Mediated moderation indirect effects.

		Outcome Variables					
		Approach Crafting			Avoidance Crafting		
Interactions	Mediators	Estimate	S.E.	99% CI	Estimate	S.E.	99% CI
GAIA × INCSR	Harmonious Passion	0.112 ***	0.040	[0.032, 0.236]	0.075	0.039	[−0.014, 0.197]
GAIA × EXCSR	Obsessive Passion	−0.002	0.012	[−0.040, 0.030]	0.085 ***	0.031	[0.012, 0.184]

Note: N = 316. GAIA represents GenAI awareness, INCSR represents perceived internal CSR, EXCSR represents perceived external CSR. *** *p* < 0.001.

## Data Availability

The dataset supporting the conclusions of this article is available in the Mendeley Data repository: https://data.mendeley.com/datasets/fn4snf9hcj/1 (accessed on 19 May 2025).
